# Development of recombinant nucleocapsid protein-based indirect enzyme-linked immunosorbent assay for sero-survey of porcine reproductive and respiratory syndrome

**DOI:** 10.14202/vetworld.2020.2587-2595

**Published:** 2020-12-05

**Authors:** S. Phani Kashyap, Jagadish Hiremath, S. Vinutha, Sharanagouda S. Patil, Kuralayanapalya P. Suresh, Parimal Roy, Divakar Hemadri

**Affiliations:** 1 ICAR-National Institute of Veterinary Epidemiology and Disease Informatics, P.B. No. 6450, Yelahanka, Bengaluru, Karnataka, India; 2 Department of Microbiology and Biotechnology, Jain University, Bengaluru, Karnataka, India

**Keywords:** indirect enzyme-linked immunosorbent assay, nucleocapsid protein, porcine reproductive and respiratory syndrome, sensitivity, specificity

## Abstract

**Background and Aim:**

Porcine reproductive and respiratory syndrome (PRRS) is a disease endemic in many countries and is of economic importance. India was free from PRRS until the first outbreak was reported from a North-East Indian state in 2013. Since then, disease outbreaks have been reported from North-East India and the pilot study conducted earlier showed that it is gradually spreading to the rest of India. Considering there are no locally developed population screening tests available for PRRS and imported diagnostic/screening tests are expensive, the present study was aimed at developing recombinant nucleocapsid (rN) protein-based indirect enzyme-linked immunosorbent assay (iELISA).

**Materials and Methods:**

The rN protein of PRRS virus (PRRSV) was produced following standard cloning, expression, and purification procedures. Using this antigen, iELISA was optimized for the detection of serum antibodies to PRRSV. The sensitivity and specificity of the test were assessed by comparing it with a commercial PRRSV antibody detection kit.

**Result:**

A total of 745 serum samples from ten different states of India were screened using the developed iELISA. The iELISA had a relative specificity of 76.18% and sensitivity of 82.61% compared to the commercial ELISA (Priocheck PRRSV ELISA kit, Thermo Fisher Scientific, USA).

**Conclusion:**

The iELISA, which deployed rN protein from Indian PRRSV, was found to be suitable in the serological survey and may be a useful tool in future disease surveillance programs.

## Introduction

Porcine reproductive and respiratory syndrome (PRRS) is an economically important and serious pig disease characterized clinically by reproductive failure in sows and respiratory disease in pigs of all ages. Delay in return to estrus and increased pre-weaning mortality is common, resulting in higher economic loss to pig farmers [1]. The disease is caused by the PRRS virus (PRRSV), with two species recognized within it [2]; *Betaarterivirus suid 1* (PRRSV-1, European genotype represented by Lelystad virus) and *Betaarterivirus suid 2* (PRRSV-2, North American genotype represented by ATCC VR-2332). Among the two species, PRRSV-2 is widely circulating in most Asian countries [3-5]. The genome of the virus is a single-stranded positive-sense RNA, 15kb in length that contains 11 known open reading frames (ORFs). The ORF1a and ORF 1b encode nonstructural proteins, while ORFs 2-7 encode structural proteins [6]. Major structural proteins are the nucleocapsid protein (N, 15kDa), the membrane protein (M, 19kDa), and the envelope glycoprotein (GP5, 26kDa), which are encoded by ORF 7, ORF 6, and ORF 5, respectively. The three additional glycoproteins GP2, GP3, and GP4 represent minor structural proteins [7].

The gold standard for diagnosis of PRRSV disease can be achieved through viral isolation in either primary cell or cell lines [8]. Serological methods such as enzyme-linked immunosorbent assay (ELISA) and molecular diagnostic assays such as conventional and real-time reverse transcription-polymerase chain reaction (RT-PCR) are the other most employed diagnostics worldwide [9]. Assays such as Western blot [10-12], indirect immunofluorescence assay, and immunoperoxidase monolayer assay have been used on a limited scale. The PCR based diagnostic assays are useful for the rapid detection of the agent during an outbreak situation due to their higher sensitivity; however, these may not be appropriate for population survey as these assays need specialized instruments, skilled manpower, and are not cost-effective [13-16]. Alternatively, serology-based tests are not appropriate for the early stage of infection, especially within 7 days post-infection (DPI) because PRRSV-specific antibodies are usually produced after 7 DPI [17]. Nevertheless, serological surveillance in unvaccinated populations is often the most effective and efficient methodology to assess the disease status. Among the serological tests, ELISA is the most suitable test for the detection of PRRSV antibodies in many samples because of its low cost, rapidness, and ease of performing.

The first described outbreak of PRRS in India was reported on June 26, 2013, in the state of Mizoram bordering Myanmar [18] and few more outbreaks were recorded in subsequent years [19]. A pilot-scale study by our group showed that the disease is not restricted to North-East India, but may also be present in other states as well [20]. Considering the economic importance of the disease and also that no serology-based tests are available for large scale screening of the susceptible population in India, here we report the development of an indirect ELISA (iELISA) with recombinant nucleocapsid (rN) protein as coating antigen. The nucleocapsid (N) protein was chosen because it is one of the three major structural proteins that form PRRSV virion. It is an abundant protein, which is highly antigenic, thus making it an appropriate candidate for the detection of the virus-specific antibodies for diagnostic or surveillance purposes [21]. Furthermore, among the many specific antibodies produced by PRRSV infection of pigs, the earliest produced are those directed against the nucleocapsid protein N, followed by antibodies against the membrane protein M, while antibodies to envelope protein GP5 are produced last. In addition, the antibody response to N and M proteins is stronger than that of the GP5 protein [22].

## Aim of the study

This study aimed to develop rN based iELISA for population survey of PRRS in India.

## Materials and Methods

### Ethical approval

The study did not involve the use of live animals and hence ethical approval was not required.

### Study period and location

The study was conducted at ICAR-National Institute of Veterinary Epidemiology and Disease Informatics, Karnataka, from 2016 to 2019.

### Selection of a target gene and *in silico* protein characterization

**Figure-1:**
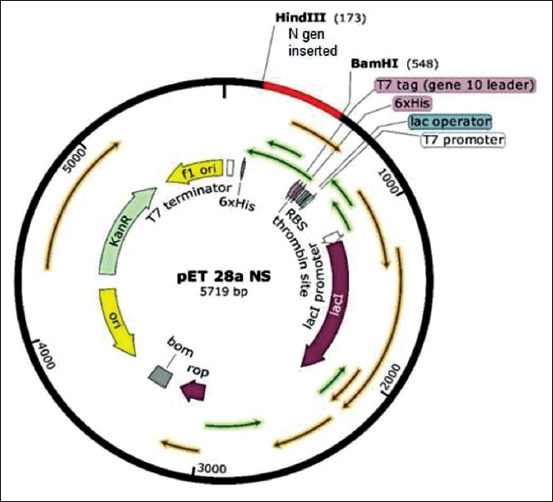
Porcine reproductive and respiratory syndrome virus (PRRSV) nucleocapsid protein clone construct with the pET28a vector. The figure describes the full length of nucleocapsid protein PRRSV genotype 2 with hexahistidine tag at N and C terminals.

The target gene, regions, and overall design of plasmid construct for the expression of nucleocapsid protein are schematically depicted in Figure-1. Initially, a representative nucleotide sequence of the entire *N* gene of PRRSV was obtained from the GenBank database at the National Center for Biotechnology Information website. Online bioinformatics tools were utilized to analyze the physicochemical properties of the target gene.

### RNA extraction, RT-PCR, and cloning

Viral RNA was extracted from a field tissue sample from Mizoram, India, using the QIAmp Viral RNA Mini Kit (Qiagen, Germany) following the manufacturer’s instructions. The RNA was reverse transcribed using the random hexamers (Qiagen, Germany) and RevertAid Reverse Transcription Kit (Thermo Scientific, USA) as described by the manufacturer. The resulting cDNA was used for PCR amplification of *ORF7* gene by Phusion High-Fidelity DNA Polymerase (New England Biolabs, UK) using a forward primer, *FP_BamHI*,_(5’ GGATCC ATG CCA AAT AAC GGC AAG C 3’), and a reverse primer, *RP_HindIII*, (5’ AAGCTT TGC TGA GGG TGA TGT GG 3′). PCR was performed under the following conditions: Initial denaturation at 98°C for 10 min, followed by 35 cycles of 98°C for 30 s, 65°C for 30 s, 72°C for 30 s, and a final extension at 72°C for 10 min. The resultant PCR product was extracted after agarose gel electrophoresis and purified using the QIAquick Gel Extraction Kit (Qiagen, Germany). The purified DNA fragment was ligated into the pJET1.2/blunt end cloning vector (Thermo Scientific, USA) and the presence of the insert in resulting plasmid was confirmed by nucleotide sequencing.

Next, the pJET1.2 vector (Thermo Scientific, USA) containing *PRRSV-N* gene, as well as a prokaryotic expression vector, pET28a (Novagen, USA) were digested separately with restriction enzymes *BamHI* and *HindIII*. Both the released *N* gene and linearized expression vector pET28a (+) were purified by gel extraction and ligated using DNA ligase (New England Biolabs, UK) at 4°C with overnight incubation to obtain recombinant plasmid. The recombinant plasmid was used for initial transformation into *Escherichia coli* TOP10 cells (Novagen, USA) and recombinant cells were selected by plating on Luria Bertani agar containing kanamycin (50 μg/mL). Single recombinant isolated cell colonies were screened by colony PCR for the presence of the insert and identity of which was confirmed by restriction endonuclease (RE) analysis using *BamHI* and *HindIII* enzymes. The purified recombinant plasmid was used for transformation into expression host, *E. coli* BL21-CodonPlus [DE3]-RIPL cells (Novagen, USA), which was grown under the selection of antibiotics, kanamycin (50 μg/mL), and chloramphenicol (35 μg/mL). Single transformed colonies with recombinant vector were screened by colony PCR for the presence of the insert, the identity of which was confirmed by RE analysis as mentioned above.

### rN protein expression

After conducting the initial expression study on a pilot scale, 1000 mL of super broth (HiMedia) medium containing kanamycin (50 μg/mL) and chloramphenicol (35 μg/mL) was inoculated with 5 mL of overnight culture and incubated for approximately 2.5 h at 37°C on a horizontal shaker. When the optical density (OD) at 600 nm reached 0.5, the expression of recombinant protein was induced by the addition of isopropyl-D-thiogalactopyranoside (IPTG) at a final concentration of 1 mM. After 3 h of incubation, 1 mL of aliquot was collected for quantitative and qualitative analysis on 15% sodium dodecyl sulfate-polyacrylamide gel electrophoresis (SDS-PAGE). The remaining bacterial culture was harvested by centrifugation at 5000 rpm for 5 min and the pellet was re-suspended in 20 mL of lysis buffer (150 mM NaCl, 200 mM Na2HPO4, 10 mM Tris-HCl, 10 mM imidazole, 6 M guanidine hydrochloride, pH 8.0) (19) containing lysozyme (1 mg/mL), Triton X-100 (0.01%), and PMSF (1 mM). After incubation at 37°C for 30 min in a shaker incubator at 220 rpm, the re-suspended pellet was lysed by sonication and repeated freeze-thawing (~2-3 times). The cell lysate was centrifuged at 12,000 rpm for 30 min at 4°C to separate soluble and insoluble fractions.

### Purification of rN protein

The supernatant containing soluble rN protein was further purified by immobilized metal ion affinity chromatography using Ni-NTA SuperFlow cartridges (Qiagen, Germany). Briefly, the supernatant was passed through Ni-NTA SuperFlow cartridge and washed with 50 mL of wash buffer (150 mM NaCl, 200 mM Na_2_HPO_4_, 10 mM Tris-HCl, 20 mM Imidazole, and 6 M guanidine hydrochloride, pH 8.0). Refolding was done by sequentially decreasing the guanidine hydrochloride by 2M each time to 0M in the buffer (pH 8.0) containing 150 mM NaCl, 200 mM Na_2_HPO_4_, 10 mM Tris-HCl, and 30 mM imidazole. Finally, the protein was eluted with 20 mL elution buffer (150 mM NaCl, 200 mM Na2HPO4, 10 mM Tris-HCl, and 500 mM imidazole, pH 8.0). The eluted protein was checked for its integrity and purity on 15% SDS-PAGE. Finally, peak fractions of eluted rN protein were pooled and dialyzed in a chilled dialysis buffer (20.0 mM Tris-Cl, 200mM NaCl, 10% glycerol, 0.5 M sucrose, and 2% glycine, pH 7.5) to renature/refold the protein.

### Protein quantification and characterization

The solubilized protein was quantified using Pierce Modified Lowery Protein Assay Kit (Thermo Scientific, USA). The reactivity of protein established by Western blot using Penta His Antibodies (Qiagen, Germany), as well as with PRRSV-specific antibodies in two separate tests. The binding of recombinant protein and the antibodies were visualized using anti-pig horseradish peroxidase (HRP, Thermos Scientific, USA) conjugate and 3,3′-diaminobenzidine substrate. The final protein solution after quantification and characterization was stored at −80°C for further analysis.

### Optimization of reagent dilutions

To determine the optimum coating concentration of recombinant protein and optimum serum dilution, chequered board titration was performed. The recombinant protein was serially diluted starting form the highest concentration of 1000 ng/well to the lowest concentration of 0.45 ng/well in 0.1 M carbonate-bicarbonate coating buffer (pH 9.6), in a 96-well flat-bottom maxi binding immunoplate (SPL Life Sciences, Korea) and incubated overnight at 4°C. The reference positive and negative sera used in the study were collected from the field that was tested positive or negative for anti-PRRSV antibodies by commercial ELISA kit (Priocheck PRRSV ELISA kit, Thermo Fisher Scientific, USA). The samples were diluted serially from 1 in 2 to 1 in 128. Signal-to-noise (S/N) ratio (S/N ratio=mean OD of positive reference/mean OD of negative reference) calculated from the OD values obtained after the end of the test was plotted against the antigen dilution for determination of ideal antigen and serum dilution. Using the optimized values of coating antigen and serum dilution, the optimum dilution of goat-anti-pig HRP conjugate was determined by serial dilution from 1:5000 to 1:20,000. All the experiments were repeated in triplicate.

### Development of the iELISA

The purified rN protein was coated onto the flat bottom, 96 well, maxi binding immunoplate (SPL Life Sciences, Korea) at the predetermined concentration in 0.1 M carbonate-bicarbonate buffer pH 9.6 (Sigma-Aldrich, USA) and incubated overnight at 4°C. After the washing step, the coated wells were subsequently blocked with 300 μL of phosphate-buffered saline with Tween 20 (0.1%, PBST) containing 5% (w/v) skimmed milk powder (HiMedia, India), 2% (w/v) bovine serum albumin (HiMedia, India), and 3% (w/v) gelatin (Difco, USA) for 1 h at 37°C. After the washing step, the test serum samples at a predetermined dilution in the blocking buffer were added in duplicates (50 μL/well) and incubated for 60 min at 37°C. Each plate also contained positive, negative reference sera, and no sample control in duplicates. After the incubation step, the plates were washed 3 times with PBST, and 50 μL of the anti-pig immunoglobulin HRP conjugate with a dilution of 1:10,000 in blocking buffer was added into each well and incubated for 60 min at 37°C. After washing, 50 μL of substrate solution (0.5 mg/mL of o-phenylene diamine dihydrochloride in phosphate citrate buffer [Sigma, USA] pH 5.0 containing 0.03% H_2_O_2_) was added per well and incubated for 15-20 min at 37°C. The reaction was stopped by the addition of 1.5M H_2_SO_4_ to the plate. The OD values were measured at 492 nm with a reference at 620 nm. The test was run for 5 days consecutively for repeatability experiments by the same technician using a set of samples (25 negative and 20 positive samples), the status of which was determined by the commercial ELISA.

### Estimation of cutoff and evaluation of diagnostic sensitivity (DSn) and specificity of iELISA

To establish a negative and positive cutoff value for this test, 789 pig serum samples of unknown status drawn from various parts of the country were run in parallel with the commercial ELISA. The receiver operating characteristic (ROC) curve implemented in the MedCalc 13.2.2.0 (MedCalc Software, Mariakerke, Belgium) was used for determining the said cutoff value. The status of the samples was set according to the commercial ELISA testing results. The alpha level was set at 0.05 and a 95% confidence interval (CI) for calculation. The level of agreement between the two ELISAs was determined on the sample to positive ratio for each sample using the trial version of the SPSS software (https://www.ibm.com/analytics/spss-statistics-software).

## Results

### Construction of cloning/expression of nucleocapsid (*N*) gene

The recombinant plasmid, pET28a-N, was constructed using an amplified *ORF7* gene (381 bases) with RE sites for *HindIII* on 5’ end and *BamHI* on 3’end. The gene was ligated into the plasmid in the required direction and polarity to the *lac I* promoter (Figure-1). The insertion site in plasmid has provided a hexahistidine tag on both the terminals (5’ and 3’) of the inserted gene. The presence of the cloned gene in the recombinant plasmid was verified by RE analysis and nucleotide sequencing.

**Figure-2:**
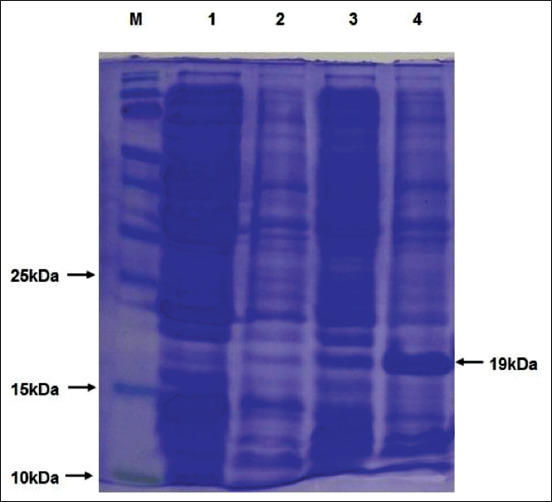
Analysis of expression of porcine reproductive and respiratory syndrome virus nucleocapsid protein by sodium dodecyl sulphate–polyacrylamide gel electrophoresis. M, Molecular weight protein marker, 1=Untransformed Escherichia coli lysate, 2=Uninduced E. coli lysate at 1 h, 3=Uninduced E. coli lysate at 3 h, 4=Induced E. coli cell lysate showing expressed rN protein (~19 kDa).

**Figure-3:**
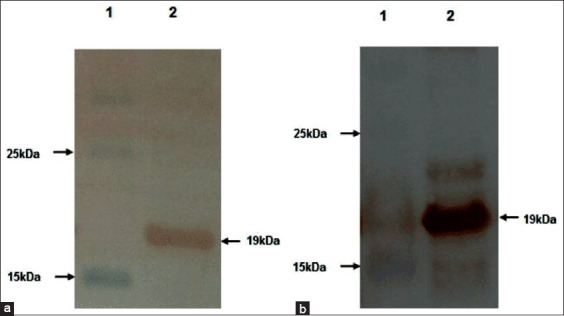
Western blot analysis of expressed recombinant nucleocapsid protein showing reactivity with (a) horseradish peroxidase-conjugated anti-His-tag monoclonal antibody (lane 2) and (b) porcine reproductive and respiratory syndrome virus positive pig serum (lane 2). Lane-1 is having protein marker in both the figures.

The 19kDa rN protein with 6 his-tag on either end was successfully expressed in *E. coli* strain BL21-CodonPlus (DE3)-RIPL (Figure-2). Since the expressed recombinant protein was mostly found in the insoluble fraction, the extraction and purification of rN protein were done under denaturing conditions. Other expression parameters such as inducer concentration and duration of induction were also optimized. The yield of recombinant protein was maximum at 1.0 mM IPTG concentration and 3 h post-induction. The protein expression was confirmed by 15% SDS-PAGE, followed by Western blotting using monoclonal antibodies against 6-His-tag (Figure-3a) and PRRSV positive field serum (Figure-3b). The results were conclusive of the successful expression of rN protein.

### Purification of rN protein

**Figure-4:**
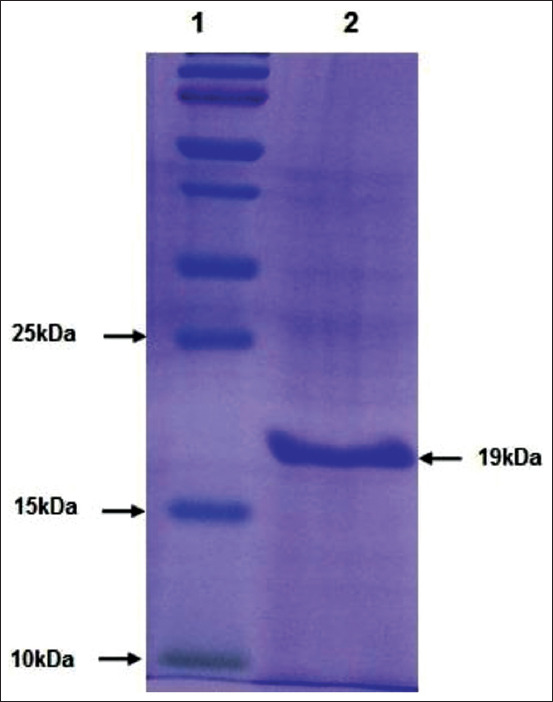
Sodium dodecyl sulfate–polyacrylamide gel electrophoresis analysis of affinity chromatography purified recombinant nucleocapsid protein. Lane-1=Protein molecular weight markers, Lane-2=Purified recombinant nucleocapsid protein.

The expressed rN protein was purified (Figure-4) initially using denaturing lysis buffer, followed by treatment with different buffers to facilitate the refolding of denatured rN protein. The protein did not precipitate during refolding, whereas dialysis of refolded protein resulted in the protein precipitating. The solubility of recombinant protein during and after dialysis was achieved by adding chemical chaperons (20.0 mM Tris-Cl, 200 mM NaCl, 10% glycerol, 0.5 M sucrose, and 2% glycine, pH 7.5) into dialysis buffer (150 mM NaCl, 200 mM Na_2_HPO_4_, 10 mM Tris-HCl, and 500 mM Imidazole, pH 8.0). The yield of this purified recombinant protein was in the range of 50-60 mg/L.

### Development and optimization of indirect ELISA (iELISA)

**Figure-5:**
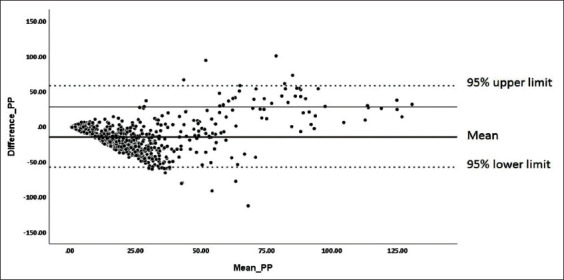
Bland-Altman plot depicting the level of agreement between the commercial enzyme-linked immunosorbent assay (ELISA) and recombinant nucleocapsid protein indirect ELISA based on the normalized optical density (percent positive) values.

**Figure-6:**
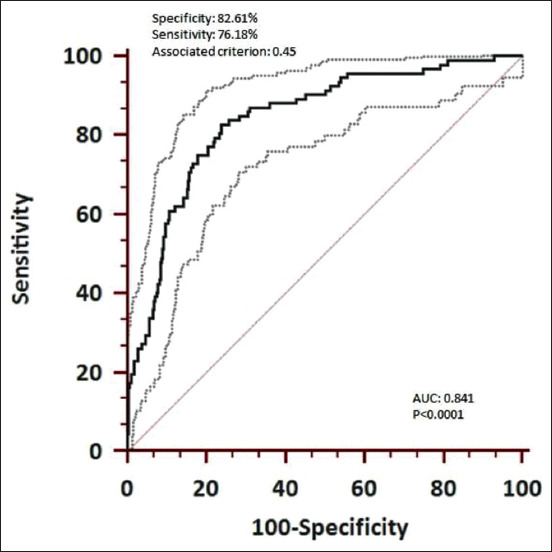
Receiver operating characteristics curve for recombinant nucleocapsid protein indirect enzyme-linked immunosorbent assay. The gray dotted lines indicate 95% confidence limits.

The rN protein-based iELISA was optimized for the detection of PRRSV specific antibodies in the swine sera. The optimal conditions for the iELISA were determined by chequered board titration. To reduce non-specific binding, the NaCl concentration in wash buffer (1× PBS) was increased to 350 mM and Tween-20 to 0.1%. The highest reactivity and lowest cross-reactivity were observed at 1:8 serum dilution, 62.5 ng/well of antigen concentration, and 1:10,000 secondary antibody (conjugate) dilution. Under the above-defined conditions, the OD of reference negative serum samples were ranged between 0.1 and 0.2 and the OD reference positive serum ranged between 1.3 and 1.5. No change in OD values was noticed for negative and positive reference serum samples after repeating the experiment thrice with the optimized parameters. To determine the cutoff value, a total of 789 serum samples received from various states of India were subjected to the detection of the PRRSV specific antibodies by Priocheck ELISA kit and the iELISA in parallel. The raw OD values obtained from both tests were normalized by percent positive (PP) ratio and agreement between them was plotted using Bland-Altman plot, which showed the mean bias±SD between first and second PP levels as −14.7957±21.91 and the limits of agreement were 28.15 and −57.74 (Figure-5). The results obtained for all the above samples in the commercial ELISA were further subjected to ROC analysis with adjusted OD values (raw OD-no sample control OD) obtained for the same samples in the iELISA. The cutoff point was determined at a criterion of OD value of 0.4566 using receiver operator characteristic (ROC) curve (Figure-6) and the area under the curve (AUC) was found to be 0.841 (95% CI 0.814-0.866, p<0.0001) with a standard error of 0.0219. The Youden index was 0.5879.

### Evaluation of the DSn, diagnostic specificity (DSp), and validation of the iELISA

Using the above-determined cutoff-value, the pig serum samples (n=789) were classified as either positive or negative and the results obtained (Table-1) were used for determining the DSn and DSp of the newly developed iELISA, which were found to be 82.61% (95% CI 73.3-89.7) and 76.18% (95% CI 72.8-79.3), respectively. The positive predictive value was found to be 31.4%, while the negative predictive value was 97.07%. The iELISA was validated on 745 purposive serum samples collected from ten states of India, of which 633 were negative, while 112 were positive. The statewide results are given in Table-2.

**Table-1:** 2×2 contingency table showing the results of rN iELISA in comparison to commercial kit.

	Commercial Kit (PrioCHECK)			Total

		Positive	Negative	
rN iELISA	Positive	76	166	242
	Negative	16	531	547
		92	697	789

rN=Recombinant nucleocapsid, iELISA=Indirect enzyme-linked immunosorbent assay

**Table-2:** State-wise results of the pig serum samples screened by iELISA.

State	Negative	Positive	Total	Percent positive
Goa	15	3	18	16.7
Karnataka	16	11	27	40.7
Madhya Pradesh	40	0	40	0
Maharashtra	26	34	60	56.7
Mizoram	97	26	123	21.1
Sikkim	46	0	46	0
Tamil Nadu	39	0	39	0
Telangana	14	3	17	17.6
Tripura	94	0	94	0
Uttar Pradesh	246	35	281	12.5
Total	633	112	745	15

iELISA=Indirect enzyme-linked immunosorbent assay

The specificity of iELISA was further examined using positive sera containing antibodies to other respiratory viral pathogens (porcine circovirus type 2 and classical swine fever) and no cross-reactions were found as all the OD values were below the cutoff point.

## Discussion

PRRS is an emerging viral disease of pigs in India that results in high economic loss to pig farmers. The economic impact of the disease is more significant among the Indian farmers, as pig farming is largely dominated by under-served members of society. In India, the disease was reported first in the North-East region of the country and a pilot study conducted by our group shows it has spread to many parts of the country. However, the estimates of disease burden, in terms of prevalence in pig populations are yet to be understood and population screening tests are essential to carry out such activities. The IDEXX-ELISA and PrioCHECK Porcine PRRSV Ab Strip Kits are commonly used as commercial PRRS ELISA kits, but they are expensive, thereby limiting their use to diagnosis. Large-scale surveys need economical, simple, and affordable tests. Therefore, the rN protein expressed in the prokaryotic system in the current study was used to develop and standardize an indirect ELISA, which could facilitate large-scale sero-epidemiological surveys to estimate the disease burden and also to understand the disease epidemiology. Based on the tentative cost estimate, it can be said that locally developed ELISA is at least 7 times less expensive than the commercial ELISA.

Toward this end, the test was optimized at a coating antigen concentration of 1.24 μg/mL which is less compared to the 2 μg/mL optimized by Chu *et al*. [22]. Recombinant fusion protein of nucleocapsid and the C-terminal 78 aa of Gp5 was optimized to 100 ng/wel [23] suggesting higher antigenicity of the recombinant protein expressed in the current study. The analytical specificity was ascertained by cross-reactivity testing, which confirms that iELISA is PRRSV specific.

In the present study, the cutoff has been determined using the ROC curve. The total area under the ROC curve is a single index for measuring the performance of a test and it was found to be 0.841 in the newly developed iELISA indicating the usefulness of the test. It is generally agreed that the larger the AUC, the better the overall performance of the diagnostic test to correctly differentiate diseased and non-diseased animals [24]. Basically, the ROC curve shows the trade-off between the true positive (sensitivity) and false-positive (1-specificity) fractions. The choice of higher specificity or sensitivity depends on situational demand. In our country, PRRS is considered as an emerging disease and the current requirement is to detect every possible case (area with cases) so that a proper containment and control plan are put in place. Keeping this in mind the cutoff point (0.45) was chosen, although this has led to the detection of a higher number of false-positive cases as compared to PrioCHECK Porcine PRRSV Ab Strip Kit. We could not use the empirical method of choosing the cutoff (mean OD of the negative controls plus 2 or 3 standard deviations) as it resulted in even higher false positives and therefore, the idea was abandoned. The PrioCHECK Porcine PRRSV Ab Strip Kit has been shown to have 100% sensitivity and 95.1% specificity [25] and has been implicated to detect more false-positive cases [25,26]. Therefore, the estimated relative specificity (76.18%) and sensitivity (82.61%) for the locally developed test may appear less than the commercial ELISA, may actually be slightly higher. Nevertheless, the performance of the locally developed test needs to be improved. The newly developed iELISA can be used for identifying hotspot regions/locations when large scale, countrywide surveys are conducted, while commercial ELISA may be used for identifying specific farms within the hotspot region to cut the cost. This proposal would address the situational demand for detecting every positive animal possible. In addition, since the sensitivity and specificity of the test are available for iELISA, the calculation of true prevalence should not be a problem. The test was validated by screening a total of 745 serum samples collected from ten different states of the country, and results were found to be satisfactory in comparison to the limited serological data available (data not are shown). Another limitation of the test was our inability to use foolproof reference serum. It is always better to use a panel of positive reference serum whose identity has been validated by clinical diagnosis as well as by virus isolation and virus neutralization. Similarly, a panel of negative reference serum collected from healthy animals with a history of no previous infection that is validated by virus neutralization or an equivalent gold standard test is important to get an unequivocal assessment of the performance of the test. Unfortunately, this could not be done due to the constraints of live virus handling. Therefore, in our study, we have used the commercial ELISA (PrioCHECK PRRSV Ab porcine) as a gold standard test to compare the performance of the iELISA. Importantly, it is to be kept in mind that the antigen used in the commercial test is derived from PPRSV-1; however, it is PRRSV-2 (*Betaarterivirus suid 2*) that is widely circulating in most of the Asian countries, including India. It has been reported that [27,28] ELISAs with coated antigens of PRRSV-1 or PRRSV-2 preferentially, but not exclusively, detect the antibodies of the respective type. The variation in the sequence length of N protein of both species, particularly as seen in PRRSV-1, can affect C-terminal nucleocapsid protein regions, which are thought to be crucial for protein folding and structure [29,30]. Using the ORF7 proteins of the PRRSV-1 field strains H2, Vas and Zap, and PRRSV-2 field strain US7, Stadejek *et al*. [31] have observed that even relatively minor differences between proteins of various strains are sufficient to affect the reactivity of swine antibodies. Taken together, the lower specificity and sensitivity as perceived in our iELISA, wherein rN protein of PRRSV-2 was used, need not be the true representation of its performance considering that there is a need to explore the impact of nucleocapsid sequence diversity on antigenicity to ensure optimal seroDSn [32].

## Conclusion

The rN protein-based iELISA developed and standardized is sufficient to detect antibodies specific to PRRSV in pig serum samples. Given the situational demand (and the conclusion that tests giving more false-negative results are more dangerous) the slightly lower sensitivity and specificity obtained in the iELISA may be more useful until any new tests can be made available. Therefore, the newly developed iELISA can be used for identifying hotspot regions or locations when large scale, countrywide surveys are conducted, while commercial ELISA may be used for identifying specific farms within the hotspot region to lower the cost. On the whole, we present the iELISA developed in this study can be used for a large serological survey as it is economical compared to commercial assay kits.

## Authors’ Contributions

SPK, DH, and JH conceived and designed the experiments. DH wrote the manuscript, while JH, SSP, KPS, and PR assisted in writing and revision of the manuscript. SPK and SV performed the experiments. All authors read and approved the final manuscript.
